# Associations between type III interferons, obesity and clinical severity of COVID-19

**DOI:** 10.3389/fimmu.2025.1516756

**Published:** 2025-04-22

**Authors:** Dana Alalwan, Alejandro Abner Garcia Leon, Gurvin Saini, Colette Gaillard, Riya Negi, Camille Heckmann, Grace Kenny, Eoin Feeney, Aoife G. Cotter, Christine Kelly, Michael Carr, Eoghan de Barra, Obada Yousif, Mary Horgan, Corinna Sadlier, Alan Landay, Gabriel Gonzalez, Patrick W. G. Mallon

**Affiliations:** ^1^ Centre for Experimental Pathogen Host Research (CEPHR), University College Dublin, Dublin, Ireland; ^2^ Université Côte d’Azur, Nice, France; ^3^ Department of Infectious Diseases, St. Vincent’s University Hospital, Dublin, Ireland; ^4^ Department of Infectious Diseases, Mater Misericordiae University Hospital, Dublin, Ireland; ^5^ National Virus Reference Laboratory, University College Dublin, Dublin, Ireland; ^6^ International Collaboration Unit, Research Centre for Zoonosis Control, Hokkaido University, Sapporo, Japan; ^7^ Department of Infectious Diseases, Beaumont Hospital, Dublin, Ireland; ^8^ Department of International Health and Tropical Medicine, Royal College of Surgeons in Ireland, Dublin, Ireland; ^9^ Endocrinology Department, Wexford General Hospital, Wexford, Ireland; ^10^ Department of Infectious Diseases, Cork University Hospital, Cork, Ireland; ^11^ University of Texas Medical Branch at Galveston, Texas, TX, United States; ^12^ Institute for Vaccine Research and Development, Hokkaido University, Hokkaido, Japan

**Keywords:** SARS-CoV-2, COVID-19, interferons, IFNλ2, IFNλ4, obesity

## Abstract

**Introduction:**

Severe COVID-19 is characterized by hyperimmune host responses contributing to airway damage and acute respiratory distress syndrome. Type III interferons (IFN), including IFN lambda 4 (IFNλ4), expressed in individuals harboring the rs368234815-ΔG allele, are implicated in host immune responses to viral infections, including SARS-CoV-2.

**Methods:**

We investigated associations between IFNλ4 expression through genotyping and COVID-19 disease severity in 853 laboratory-confirmed SARS-CoV-2 cases enrolled in the All-Ireland Infectious Diseases Cohort. Additionally, we measured plasma levels of Type I, II and III IFN using quantitative immunoassays along with IFNλ4 expression and COVID-19 disease severity in a sub-group [n=321 (37.6%)] with samples available within 10 days of symptom onset. IFNλ4 was expressed in 382 (44.8%) but expression was not significantly associated with COVID-19 disease severity.

**Results:**

Within the sub-group, we found no consistent associations between IFNλ4 expression and circulating IFNs. However, we observed significantly increased expression of IFNλ1 and IFNλ2 in severe COVID-19 (P<0.01), with IFNλ2 remaining significantly associated after adjustment for age, sex, ethnicity, and comorbidities, including obesity (BMI≥30 kg/m2) (P<0.001). Interestingly, although IFNλ2 levels were significantly higher in subjects with obesity, the association between higher IFNλ2 and COVID-19 disease severity was only observed in individuals without obesity (P<0.01).

**Conclusion:**

These data reveal an important role for IFNλ2 as an immune correlate that predicts COVID-19 disease severity, which may be masked in those with obesity.

## 1 Introduction

At the onset of the COVID-19 pandemic, despite limited genetic variation in the SARS-CoV-2 genome, some individuals developed respiratory failure, while others remained asymptomatic or experienced only mild symptoms (1), even before treatments or prophylactic vaccines became widely available. Older age and presence of clinical comorbidities such as obesity are associated with an increased risk of severe COVID-19 (2), particularly in the pre-vaccine era, possibly mediated through an impaired cellular environment resulting in distorted expression of interferons (IFNs) and an impaired antiviral immunity (3).

IFNs, a principal group of antiviral cytokines, are broadly classified into three main types based on their specific receptors and structural homologies, including IFN types I, II, and III (4). Type I IFNs include IFNα, β, ϵ, k, ω, and δ, type II comprises IFNγ (5), while type III IFNs comprise the interferon lambda (IFNλ) family and were initially termed IL-29, IL-28A, and IL-28B, but later renamed IFNλ1, 2, and 3, respectively (6).

Genome-wide association studies (GWAS) have identified single nucleotide polymorphisms (SNPs) that are associated with expression of a fourth type III IFN; IFNλ4. Together, the IFNλ family have been shown to impact on antiviral immune responses and correlate to infectious disease outcomes in HCV, HIV, CMV, and influenza viral infection (7, 8).

IFNλ4 expression is determined by two major SNPs: rs368234815 and rs117648444. The former harbors a frameshift mutation that changes the genotype from ΔG-expressing to TT-non-expressing. The ΔG genotype expression allows for the production of a functional IFNλ4 open reading frame for a functional IFNλ4 protein, while the TT allele results in a truncated non-functional protein which abolishes the expression of IFNλ4 (9). Importantly, IFNλ4 expression is found in only 50% of the world population with striking ethnic differences; 50% of Europeans, 90% of Africans, and 10% of Asian individuals (10).

The genotype of a neighboring SNP, rs117648444 (G/A: IFNλ4 P70/S70), can also modify the biological activity of rs368234815-ΔG (10). The rs117648444 activity variant SNP results in a missense mutation that gives rise to a non-synonymous mutation P70S (11). This hypomorphic rs117648444-A allele only occurs when the ΔG is present in rs368234815. The two SNPs can thus differentiate individuals into three genotypic groups: IFNλ4-null (rs368234815-TT/TT, rs117648444-G/G), IFNλ4-strong (rs368234815-TT/ΔG and ΔG/ΔG, rs117648444-G/A), and IFNλ4-weak (rs368234815-TT/ΔG and ΔG/ΔG, rs117648444-A/A) (10).

Obesity and a high body mass index (BMI) were classified as significant predictors of COVID-19 outcomes and severity (12). The relationship between inflammation and metabolism has recently become evident, as changes in metabolism, such as those seen in obesity, can lead to inflammation. Conversely, inflammation resulting from viral infections can also induce metabolic changes (13). Teran-Cabanillas et al. have demonstrated impaired interferon responses in individuals with obesity, specifically IFNα, IFNβ and IFNλ, affecting their immune function (14). This indicates the role that obesity plays in modulating immune responses to viral infections.

Dysregulated IFN responses are deemed a key factor in COVID-19 pathogenesis, but conflicting results are observed in different studies examining COVID-19 disease severity and IFN responses. Early induction of type I and III IFNs may produce a favorable outcome of COVID-19 disease progression (15). While type I IFNs act rapidly to induce an immune response via the expression of chemokines and cytokines (16), type III are considered to be tissue protective and usually lack the accompanying pro-inflammatory response induced by type I IFNs (17). However, a number of studies have also associated type I IFN responses with an increased expression of interferon stimulated genes (ISGs), proinflammatory genes, and cytokines in individuals with severe COVID-19 (18). Kwon et al, have shown inflated type I/II IFNs (IFNα and IFNγ) in severe COVID-19 within 5-10 days of symptom onset, notably IFNα tended to correlate with the viral load in their cohort (19). Conversely, other studies have shown a greatly weakened type I and III IFN response in early COVID-19 infection (20). The timing of IFN induction whether type I or III, may also be an integral part in regulating the outcome of COVID-19 disease.

Given this lack of clarity on the role of IFNs in COVID-19 infection, we sought to investigate what role, if any, expression of IFNλ4 plays in modifying disease severity in COVID-19 and additionally to explore relationships between early infection levels of other circulating type I, II, and III IFNs and COVID-19 disease severity, considering important clinical predictors such as age and presence of comorbidities such as obesity.

## 2 Materials and methods

### 2.1 Study design and participants

This analysis was conducted within the All-Ireland Infectious Diseases Cohort (AIID Cohort), a prospective, multicenter, observational cohort that recruits individuals attending hospitals in Ireland for issues relating to infectious diseases, including COVID-19. The study was approved by the National Research Ethics Committee as part of The AIID Cohort (20-NREC-COV-056), and all subjects provided written informed consent for the collection of samples and clinical data for further research. This analysis was restricted to participants with confirmed PCR-positive SARS-CoV-2 with samples collected between March 2020 and June 2021. The participants were grouped according to the WHO COVID-19 severity scale into mild, moderate, and severe/critical (21). Within the analytical group, a subgroup of biobanked samples collected within 10 days of symptom onset was selected for the analysis of early IFN responses. This timeframe was chosen to capture the critical window of early immune activation, which may occur before the onset of severe symptoms, typically observed on or after day 10.

### 2.2 Nucleic acid extraction

Genomic DNA was extracted from Biobanked buffy coats obtained from 3 mL sodium citrate blood, or cellular free DNA was extracted from plasma samples obtained from 10 mL ethylenediaminetetraacetic acid (EDTA) blood were utilized for DNA extraction using the MagNA Pure 96 instrument and large volume kit (Roche Diagnostics, Rotkreuz, Switzerland), as per manufacturer’s instructions.

### 2.3 Genotyping assay

IFNλ4 SNPs rs368234815 and rs117648444 genotyping was performed employing the TaqMan SNP genotyping assay using custom made detection mixes (Applied Biosystems, Waltham, Massachusetts, USA), as described previously (22). Briefly, the SNP genotyping assays are dependent on differently labelled fluorescent probes that discriminate the target nucleotide sequence. The VIC dye detects Allele 1 (Allele X) wild-type sequence, and the FAM dye detects Allele 2 (Allele Y) mutant sequence. Heterozygous samples contain an equal signal contribution of both FAM and VIC dyes.

### 2.4 Immunoassay biomarkers measurement

Type I IFN (IFNα2a and IFNβ), Type II IFN (IFNγ) and Type III IFN (IFNλ1) were measured using a multiplex electrochemiluminescence assay (Meso Scale Discovery, Rockland, MD, USA, Cat no. K15094K-2). IFNλ2 and IFNλ3 were measured using the Luminex MAGPIX platform (Biotechne R&D Systems, Minneapolis, MN, USA, Kit name: LXSAHM-22), results were analyzed using the Luminex xPONENT for MAGPIX software (version 4.3). EDTA plasma samples were run in duplicate alongside a calibration curve. Samples with intraplate coefficient of variance (CV) above 10% were repeated. Samples with variable CV after two repeats were excluded from further analysis.

### 2.5 Statistical analysis

We used Hardy-Weinberg analysis to test the equilibrium of the population genetics and Chi-Square χ^2^ tests with the goodness of fit with two degrees of freedom to investigate the observed and expected allele frequencies. The association of COVID-19 disease severity outcome with SNP genotypes of rs368234815 (TT/TT, TT/ΔG, and ΔG/ΔG), and between group demographic differences were analyzed using a Chi-Square χ^2^ test to compare counts of categorical variables and Kruskal-Wallis test to compare distributions of continuous variables. Measured circulating interferon concentrations were natural log transformed and we employed a stepwise multinomial logistic regression to explore the impact of clinical covariates on the relationship between IFN levels and COVID-19 disease severity, correcting for age, sex, Caucasian ethnicity, comorbidities (obesity (BMI ≥ 30 kg/m^2^), metabolic disease, and respiratory disease), and other significant IFNs associated with disease severity in univariate analysis. A P-value <0.05 was considered significant. Data are presented as median (interquartile range) unless stated. All statistical analyses were performed with IBM^®^ SPSS^®^ Statistics (version 27), RRID: SCR_016479, GraphPad Prism Software, LLC. (version 9.5.1), RRID: SCR_002798, and R (R version 2024.04.0 + 735, http://www.r-project.org), RRID: SCR_001905, using ggplot2 package version 3.1.1 (23), RRID: SCR_014601.

## 3 Results

### 3.1 Cohort demographics

Of 886 laboratory-confirmed SARS-CoV-2, AIID participants that were eligible for inclusion in the analysis, 853 had available data. Characteristics of the study population are shown in **Table 1**. Median (IQR) age was 53.8 (39.6, 66.7), 486 (56.9%) were female and the majority (78.5%) of the cohort were of Caucasian ethnicity. Participants were categorized as having mild [n=500 (58.62%)], moderate [n=164 (19.2%)], and severe [n=189 (22.2%)] COVID-19.

**Table 1:** Characteristics of overall study population.

WHO COVID-19 Severity Category	Total [n=853]	Mild [n=500 (58.62%)]	Moderate [n=164 (19.2%)]	Severe [n=189 (22.2%)]	P
Age (years) - Median (IQR)	53.8 (39.6, 66.7)	45.9 (32.6, 61.0)	58.7 (50.0, 70.5)	63.8 (53.4, 75.4)	**<0.0001**
Sex at Birth - N (%)					
Female	486 (56.9)	328 (65.6)	75 (45.7)	83 (43.9)	**<0.0001**
Male	367 (43.1)	172 (34.4)	89 (54.3)	106 (56.1)	
Ethnicity - N (%)					
Caucasian	670 (78.5)	421 (84.2)	116 (70.7)	136 (72.0)	**<0.0001**
Other	183 (21.5)	79 (15.8)	48 (29.3)	53 (28.0)	
BMI (kg/m^2^) - Median (IQR)	27.7 (24.2, 32.3)	25.9 (23.2, 31.1)	28.9 (25.4, 32.5)	30.0 (26.5, 34.3)	**<0.0001**

Characteristics of Overall Study Population. IQR, interquartile range; BMI, body mass index; WHO, World Health Organization. Chi-Square χ^2^ test to compare counts of categorical variables and Kruskal-Wallis test to compare distributions of continuous variables.

Bold means significant P values.

Of the full cohort, 321 (37.6%) participants had samples collected within 10 days of symptom onset and were included in the sub-group analysis of IFN biomarkers (**Table 2**). Within this sub-group, median (IQR) age was 62.1 (47.8, 76.7), with a majority male [n=174 (54.2%)] and of Caucasian ethnicity [n=252 (78.5%)]. Most of the sub-cohort (81.5%) reported underlying comorbidities including but not limited to hypertension [n=135 (42.2%)], diabetes [n=47 (14.7%)], obesity [n=253 (29.7%)], and respiratory disease [n=81 (25.3%)]. Median (IQR) BMI was 27.4 (24.2, 31.8) kg/m^2^. The majority (84.7%) of the cohort were admitted to hospital primarily due to SARS-CoV-2 infection.

**Table 2:** Clinicopathological features of early sampling study population.

WHO COVID-19 Severity Category	Total [N=321]	Mild [n=156 (48.6%)]	Moderate [n=70 (21.8%)]	Severe [n=95 (29.6%)]	P
**Age (years) - Median (IQR)**	62.1 (47.8, 76.7)	58.8 (33.3, 73.5)	58.7 (51.5, 73.7)	70.7 (56.0, 80.4)	**<0.0001**
Sex at Birth - N (%)					
Female	147 (45.8)	77 (49.4)	33 (47.1)	37 (38.9)	0.2666
Male	174 (54.2)	79 (50.6)	37 (52.9)	58 (61.1)	
Ethnicity - N (%)					
Caucasian	252 (78.5)	134 (85.9)	51 (72.9)	67 (70.5)	**0.0069**
Other	69 (21.5)	22 (14.1)	19 (27.1)	28 (29.5)	
**Underlying Comorbidities - N (%)**	260 (81.5)	116 (74.4)	60 (87.0)	84 (89.4)	**0.0118**
Hypertension	135 (42.2)	58 (37.2)	27 (38.6)	50 (53.2)	**0.0461**
Diabetes	47 (14.7)	19 (12.2)	13 (18.6)	15 (16.0)	0.3909
Obesity	253 (29.7)	34 (21.8)	23 (32.9)	32 (33.7)	0.0692
Respiratory Disease	81 (25.3)	25 (16.0)	25 (35.7)	31 (33.0)	**0.0010**
Renal Disease	2 (0.6)	1 (0.6)	0 (0.0)	1 (1.1)	0.6967
Immunosuppressive Conditions	14 (4.4)	6 (3.8)	1 (1.4)	7 (7.4)	0.1651
**BMI (kg/m^2^) - Median (IQR)**	27.4 (24.2, 31.8)	25.1 (23.0, 30.9)	29.4 (26.1, 32.5)	29.2 (25.7, 32.3)	**0.0006**
Hospitalization Status - N (%)					
Admitted	272 (84.7)	107 (68.6)	70 (100)	95 (100)	**<0.0001**
Outpatient	49 (25.3)	49 (31.4)	0 (0.0)	0 (0.0)	
COVID-19 Complications - N (%)					
Invasive Ventilation	15 (4.6)	1 (0.6)	0 (0.0)	14 (14.7)	**<0.0001**
Viral Pneumonia	107 (33.0)	12 (7.7)	37 (53.9)	58 (61.1)	**<0.0001**
Bacterial Pneumonia	32 (9.9)	6 (3.8)	10 (14.3)	16 (16.8)	**0.0015**
ARDS	52 (16.0)	0 (0.0)	2 (2.9)	50 (52.6)	**<0.0001**
Disease Outcome - N (%)					
Discharged	235 (73.2)	105 (67.3)	70 (100)	60 (63.2)	**<0.0001**
Death	36 (11.2)	1 (0.6)	0 (0.0)	35 (36.8)	
Outpatient	50 (15.6)	50 (32.1)	0 (0.0)	0 (0.0)	

Clinicopathological Features of Early Sampling Study Population. IQR, interquartile range; BMI, body mass index; WHO, World Health Organization; ARDS, Acute Respiratory Distress Syndrome. Chi-Square χ^2^ test to compare counts of categorical variables and Kruskal-Wallis test to compare distributions of continuous variables.

Bold means significant P values.

### 3.2 Interferon λ SNPs genotype characteristics and association with disease severity

Allelic discrimination data for the cohort (n=853) yielded rs368234815 SNP TT/TT major homozygous genotype [n=471 (55.2%)], TT/ΔG heterozygotes [n=310 (36.3%)], and ΔG/ΔG minor homozygotes [n=72 (8.4%)] respectively, with 382 (44.8%) of the cohort expressing IFNλ4 (TT/ΔG, ΔG/ΔG). The rs117648444 SNP showed 88.3% (n=753), 11.25% (n=96), and 0.47% (n=4) allelic frequencies for the three genotypes G/G, G/A, and A/A, respectively. Of those expressing IFNλ4, 312 (81.7%) had a strong IFNλ4 outcome. Hardy-Weinberg tests showed no significant departure from equilibrium for both investigated SNPs in this cohort [rs368234815 (P=0.127) and rs117648444 (P=0.884)]. The distribution of genotype allele frequencies of rs368234815 and rs117648444 SNPs according to COVID-19 disease severity group are shown in **Supplementary Table 1**. There were no significant differences in genotype frequencies between COVID-19 severity groups.

### 3.3 Circulating plasma levels of type I, II, and III interferons

**Figure 1:**
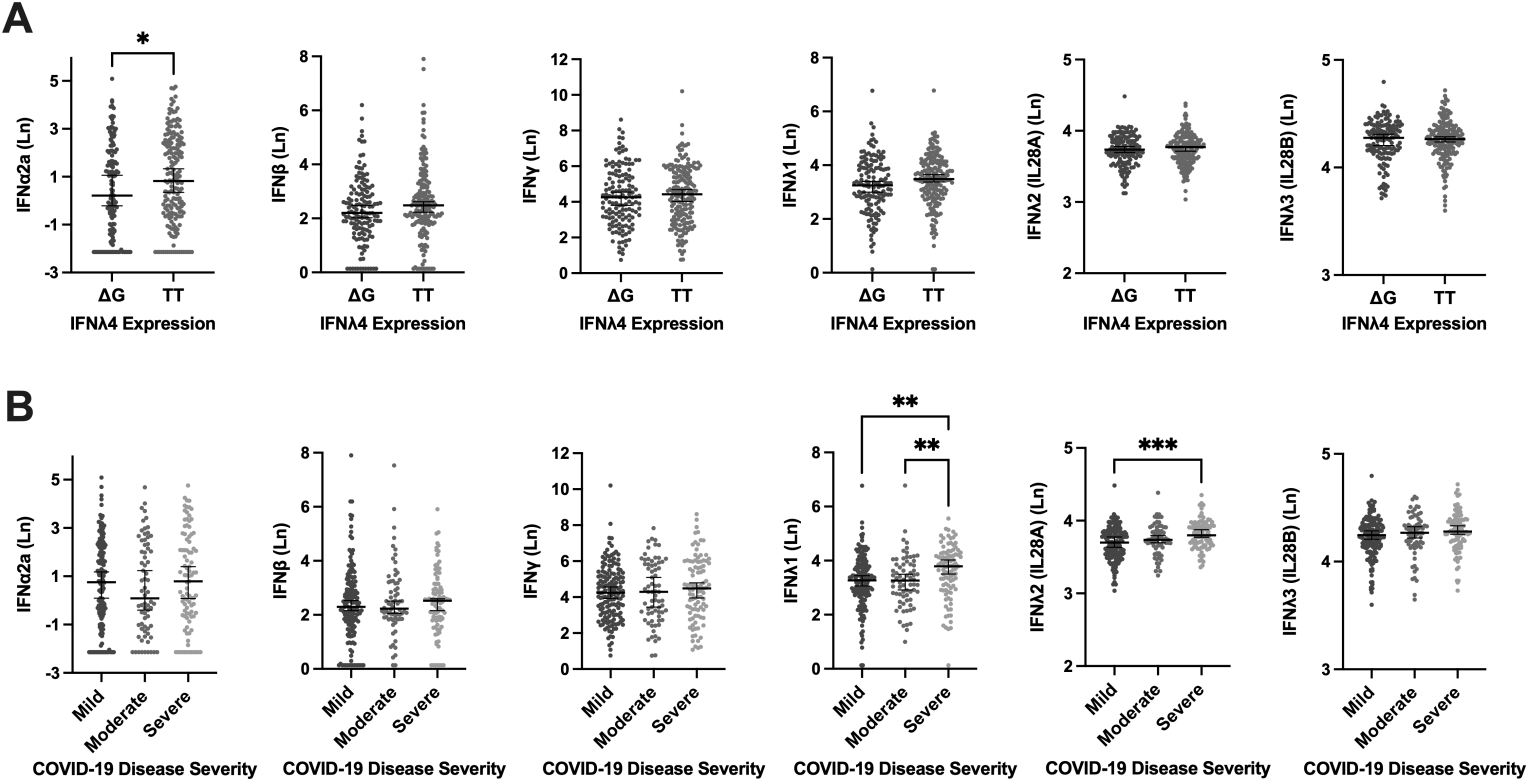
Circulating Levels of Type I (IFNα2a, and IFNβ), Type II (IFNγ), and Type III [IFNλ1, IFNλ2 (IL28A), IFNλ3 (IL28B)] Interferons Stratified by IFNλ4 Expression and COVID-19 Severity Groups. Circulating Levels of Type I (IFNα2a, and IFNβ), Type II (IFNγ), and Type III [IFNλ1, IFNλ2 (IL28A), IFNλ3 (IL28B)] Interferons Stratified by IFNλ4 Expression and COVID-19 Severity Groups in the Sub-cohort (n=321). **(A)** Assessment of the expressing (TT/ΔG, and ΔG/ΔG) and non-expressing genotypes (TT/TT) of IFNλ4 effects on the concentration of Type I, Type II, and Type III Interferons. **(B)** WHO COVID-19 disease severity criteria association with plasma concentration of Interferons. (Ln) Natural log. Error bars represent the median and interquartile range. *P ≤ 0.05, **P ≤ 0.01, ***P ≤ 0.001.

We next investigated whether type I (IFNα2a, and IFNβ) or type II (IFNγ) IFN concentrations in plasma varied with the expression of IFNλ4-rs368234815. Only IFNα2a concentrations differed, with significantly higher concentrations in those not expressing IFNλ4 (rs368234815-TT/TT) [0.819 (-0.544, 2.422) pg/mL] compared to expressing IFNλ4 rs368234815-TT/ΔG and ΔG/ΔG genotypes [0.214 (-1.278, 2.027) pg/mL] (P=0.041). Concentrations of IFNλ1, IFNλ2, and IFNλ3 did not vary between those who express and do not express IFNλ4 (**Figure 1A**).

Concentrations of type I (IFNα2a, and IFNβ) and type II (IFNγ) IFNs were not different between COVID-19 disease severity groups (**Figure 1B**). For type III IFNs (IFNλ1, IFNλ2, and IFNλ3), IFNλ1 levels were higher in those with severe COVID-19 [3.789 (3.031, 4.277) pg/mL] compared to mild [3.269 (2.747, 3.842) pg/mL] (P=0.0028), and moderate [3.288 (2.675, 3.858) pg/mL] (P=0.0096) groups. Similarly, IFNλ2 levels were also significantly higher in the severe group [3.799 (3.694, 3.932) pg/mL] compared to the mild [3.698 (3.540, 3.838) pg/mL] (P<0.001), but not different from the moderate group [3.739 (3.656, 3.872) pg/mL]. Circulating IFNλ3 levels did not vary between COVID-19 severity groups (**Figure 1B**).

### 3.4 Impact of obesity on IFN lambda and COVID-19 disease severity

**Figure 2:**
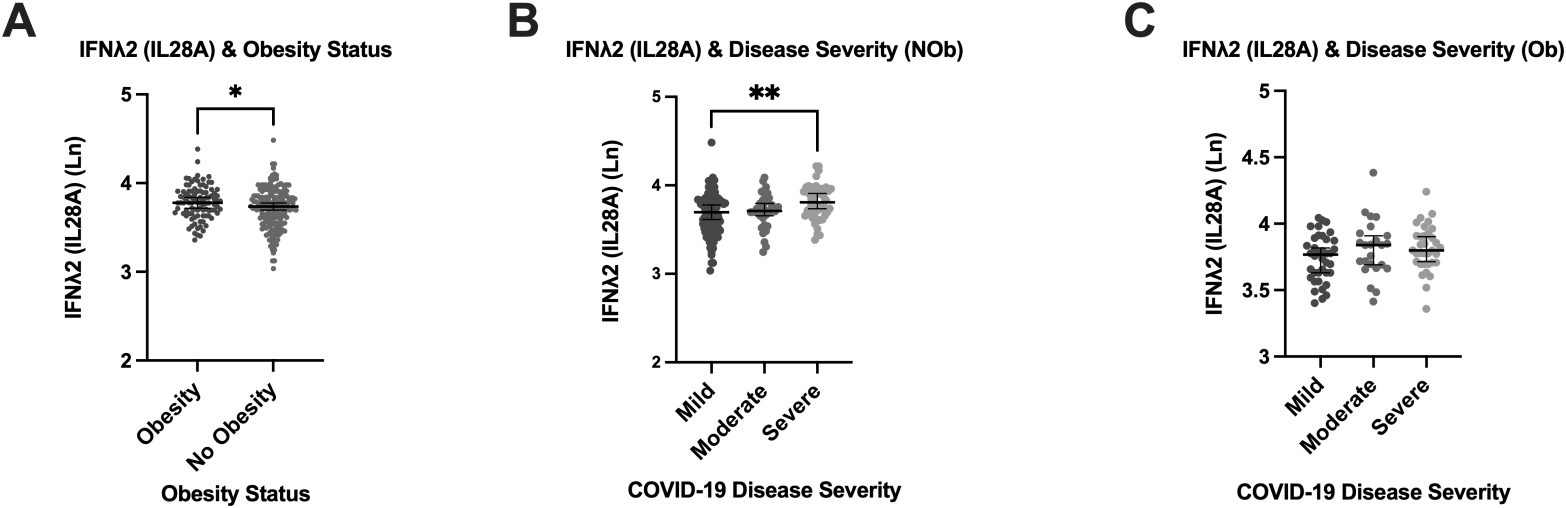
Relationship between Type III IFNλ2 (IL28A), Obesity and COVID-19 Disease Severity. Relationship between Type III IFNλ2 (IL28A), Obesity and COVID-19 Disease Severity in the Sub-cohort (n=321). **(A)** Association of obesity status with plasma concentration of type III IFNλ2 (IL28A). **(B)** Levels of type III IFNλ2 (IL28A) in the non-obese. **(C)** Levels of type III IFNλ2 (IL28A) in the obese. Obesity defined as BMI ≥ 30 Kg/m^2^. (NOb) non obese; (Ob) obese; (Ln) Natural log. Error bars represent the median and interquartile range. *P ≤ 0.05, **P ≤ 0.01.

We next investigated the association between obesity (BMI ≥ 30 kg/m²) and the induction of IFN responses across different COVID-19 severity categories, given its strong link to severe disease outcomes and related comorbidities, which may influence immune responses. The median (IQR) BMI in the mild [25.1 (23.0, 30.9)] COVID-19 group was significantly lower than the moderate [29.4 (26.1, 32.5) kg/m^2^] and the severe [29.2 (25.7, 32.3) kg/m^2^] groups (P=0.0006). Between IFNλ1 and IFNλ2, only IFNλ2 levels were significantly higher in those with obesity (**Figure 2A**), while IFNλ1 levels did not significantly differ between those with and without obesity. However, exploring associations between IFNλ2 and COVID-19 severity according to obesity group, revealed a significantly higher IFNλ2 in those with severe disease only in the people without obesity [3.808 (3.670, 3.945) pg/mL] group (P=0.0017) (**Figure 2B**), as opposed to people with obesity group [3.799 (3.696, 3.910) pg/mL] (**Figure 2C**).

**Figure 3:**
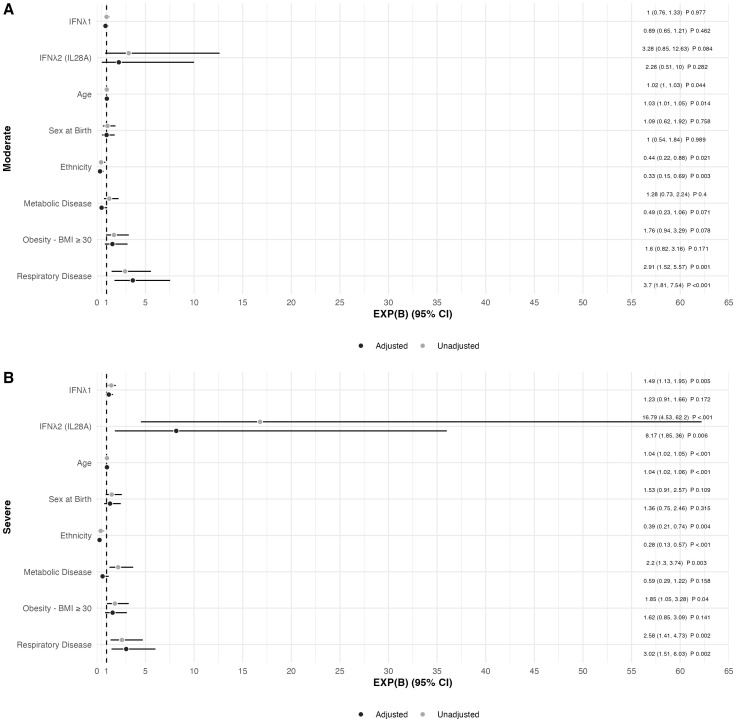
Forest Plot of Factors Associated with COVID-19 Disease Severity in the Sub-cohort (n=321) in **(A)** Moderate and **(B)** Severe COVID-19 disease adjusted for age, sex at birth, Caucasian ethnicity, obesity (BMI ≥ 30 Kg/m^2^), metabolic disease, and respiratory disease. Mild COVID-19 disease was used as reference value. Data presented as the odds ratio [EXP(B)] with the 95% confidence interval for EXP(B).

In a forward, stepwise, multinomial logistic regression exploring factors associated with COVID-19 severity, including demographics (age, sex at birth, and ethnicity), and comorbidities (obesity, metabolic disease, and respiratory disease) (**Figure 3**), IFNλ2 was the only biomarker that remained significantly associated with severe COVID-19 in fully adjusted analyses, with higher levels of IFNλ2 associated with a higher likelihood of severe COVID-19 [odds ratio [EXP(B)] (95% confidence interval)] [8.165 (1.850, 36.04)]. In addition, a history of respiratory disease, older age and non-Caucasian ethnicity were also independently associated with severe COVID-19 in fully adjusted analyses.

## 4 Discussion

This study investigated the association of early host IFN responses with COVID-19 disease severity. We demonstrate that type III IFNs, and particularly IFNλ2, may play a role in COVID-19 disease outcome. Additionally, we found higher IFNλ2 levels in those with obesity and a relationship between IFNλ2 and COVID-19 disease severity that was only observed in those without obesity, suggesting an interaction between obesity and IFNλ2 responses that could help explain differing clinical outcomes to COVID-19 in people with obesity. However, when investigating the expression of two common IFNλ4 SNP genotypes of rs368234815 and rs117648444, we found that the expression of IFNλ4 had no impact on clinical disease severity in COVID-19 in this study.

Type I and III IFNs demonstrate a paradoxical role in mediating host immune responses to SARS-CoV-2 infection, as their role in early infection is not well elucidated, with early increased levels shown to be protective against severe disease in some studies, while others have shown the opposite (24, 25). Zaleska et al. found lower levels of IFNλ2 in severe COVID-19 compared to the moderate group, where the majority of the severe group had undetectable levels of IFNλ2 (26). In contrast, our unadjusted analyses showed higher IFNλ1 and IFNλ2 levels in those with moderate and severe COVID-19, possibly driven by the high prevalence of comorbidities in our cohort, which may have amplified the type III IFN response. Nonetheless, our findings align with Ruytinx et al. who demonstrated higher IFNλ1 in severe and critically ill individuals with COVID-19, with increased IFNλ1 also associated with a higher odds ratio of ICU mortality (27). However, after adjustment, only higher IFNλ2 remained associated with more severe COVID-19 in our cohort, with the opposite observed in the cohort of Rutinx et al. As we collected our samples within a range of 10 days of symptom onset, they collected their samples within 8 days of a positive SARS-CoV-2 PCR test. Additionally, there could be intrinsic differences of the analytical methods between both studies, although this may be a small difference between methods of time of sampling or cytokine measurement, it could still expound the differences in cytokine levels between our cohort and theirs.

The relative roles of IFNs in mediating host immune responses to respiratory viruses are complex. Our data supports a role for heightened type III IFNs, precisely IFNλ1 and IFNλ2, in driving more severe COVID-19. Type III IFNs are known to be potent cytokines provoked by ISGs (28), several cohorts have found increased transcriptional ISG signatures in individuals with severe COVID-19, where these transcriptional signatures have been linked back to higher SARS-CoV-2 viral loads which may also trigger a more intense cytokine storm (29). Taken together, these data support a scenario where increased expression of ISGs drive expression of type III IFNs which then contribute to the cytokine storm characteristic of severe COVID-19.

Interestingly we also observed differential associations of IFN levels and disease severity based on subjects’ BMI. Although we observed significantly higher IFNλ2 in subjects with obesity, the relationship between elevated IFNλ2 and severe disease was only observed in people without obesity. Various cohorts have shown that obesity is a major predictor of severe COVID-19 disease (30, 31). These studies have focused largely on epidemiological data without the consideration of inflammatory markers in their analytical models. As obesity is considered to be low-grade inflammation, it is not surprising that people with obesity experience an altered expression of pro-inflammatory cytokines, with the adipose tissue being an important producer of TNFα, IL1β, and IL6 (32). Our data would support a scenario whereby individuals with obesity have pre-existing elevations of inflammatory cytokines and interferons, including type III IFNλ2 placing them at higher risk of severe disease, whilst individuals without obesity who do not mount excessive IFNλ2 responses are relatively protected against severe COVID-19. This is confirmed by several studies that found elevated levels of type III IFNs, particularly IFNλ1 in people with obesity (33, 34).

Although, the different subtypes of the IFNλ family share highly homologous amino acid (aa) sequences which likely arose through gene duplication giving rise to paralogous sequences. IFNλ1 and IFNλ2 share 81% aa, IFNλ2 and IFNλ3 share 96% aa, while IFNλ4 may be more distantly related and possesses only 28% aa identity with other IFNλ types (7). Our outcomes showed IFNλ1 and IFNλ2 were significantly associated with severe COVID-19, whereas IFNλ4 expression was not. Our findings on IFNλ4 are consistent with previous data form a smaller Spanish cohort (N=177) of mainly Caucasian subjects (35). However, our outcomes contrast with another study that analyzed IFNλ4 expression between survivors and non-survivors in an Iranian population (N=750), which observed associations between IFNλ4-ΔG/ΔG genotype and COVID-19 mortality (36). This discrepancy between these outcomes could partially be explained by IFNλ4 allele frequencies within different ethnicities, as our cohort, although larger, consisted of a primarily Caucasian population, similar to the Spanish cohort, with lower frequencies of the ΔG allele in a European population.

These data build on the growing evidence surrounding the role of IFNλ4 in modifying host responses to viral infections, some beneficial and some less so. In Hepatitis C (HCV), the rs368234815 SNP is associated with differing responses to treatment depending on the HCV genotype (improved responses in HCV genotype 1 and 4 but not genotypes 2 and 3) (37), and the spontaneous clearance of HCV in the absence of treatment (38). In people with HIV, we previously reported on associations between rs368234815-ΔG/ΔG and increasing likelihood of normalizing CD4^+^:CD8^+^ ratio in response to antiretroviral therapy (39). In contrast, presence of rs368234815-ΔG/ΔG linked to IFNλ3-rs12979860 was associated with reduced clearance of respiratory RNA viruses in Rwandan children (40). These results suggest that the impact of host expression of IFNλ4 may have differing impacts on clinical outcomes depending on the virus and type of infection. Whether IFNλ4 polymorphisms impact upon COVID-19 severity and, most importantly, the ultimate outcome following SARS-CoV-2 infection requires further investigation.

Our study does have limitations. The high proportion of Caucasians likely resulted in an under ascertainment of the ΔG allele. A larger, more diverse cohort could provide additional insights into the impact of IFNλ4 expression and COVID-19 severity and outcome. As our samples were collected between March 2020 and June 2021, we were unable to explore the impact of COVID-19 vaccination on the interaction between type III IFN expression and COVID-19 disease severity, which may change following SARS-CoV-2 vaccination. Additionally, we did not measure neutralizing type I IFN autoantibodies nor inborn errors of type I IFN, which have also been implicated in contributing to COVID-19 disease severity (41). Furthermore, during this time period, a number of distinct SARS-CoV-2 variants of concern were in circulation with differing impacts on clinical severity which may also have impacted the analyses (42). Additionally, obesity was defined as BMI ≥ 30 kg/m², which may not fully capture other measures such as percentiles or obesity grades. However, given that most participants were Caucasian, this is unlikely to have significantly impacted our findings. Our ventilation data was limited to a binary variable, with only 15 of 321 participants requiring ventilation. Similarly, as most participants were either discharged or treated as outpatients, further analysis of ventilation duration and interferon levels between survivors and non-survivors was limited. Lastly, we did not assess the predictive value of IFN levels and IFNλ4 expression. Future studies with longitudinal data are needed.

In conclusion, common IFNλ4 genotypes were not associated with COVID-19 disease severity or expression of circulating IFNs. However, both higher IFNλ1 and IFNλ2 were associated with more severe COVID-19 disease. IFNλ2 was also higher in individuals with obesity, although associations between IFNλ2 and COVID-19 disease severity were only observed in individuals without obesity, suggesting that obesity may contribute to increased risk of severe COVID-19 through increased expression of IFNλ2, potentially impacting disease progression. These findings suggest that assessing IFNλ2 could help to identify those at a greater risk of severe disease, enabling earlier interventions and improved management strategies. Future work is required to validate these results and to study the impact of vaccination on the relationships between type III IFNs and COVID-19 severity.

## Data Availability

The data that support the findings of this study manuscript can be requested from the All-Ireland Infectious Diseases Cohort Study group. However, the data can be made available on request subject to approval by a local ethics committee.

## The All-Ireland Infectious Diseases Cohort Study investigators

Mater Misericordiae University Hospital: A. Cotter, M. Horgan, E. Muldoon, G. Sheehan, T. McGinty, JS. Lambert, S. Green, K. Leamy, C. Kelly. St Vincent’s University Hospital: G. Kenny, K. McCann, R. McCann, J. O’Halloran, C. O’Broin, S. Savinelli, E. Feeney, PWG. Mallon. CEPHR: A. Garcia Leon, D. Alalwan, S. Miles, R. Negi, G. Saini, C. Gaillard. Beaumont Hospital: E. de Barra, S. McConkey, K. Hurley, I. Sulaiman, B. Jacob. University College Cork: C. Sadlier. Sligo University Hospital: B. Whelan, Our Lady of Lourdes Hospital: J. Low. Wexford General Hospital: O. Yousif. University Hospital Galway: B. McNicholas. St Luke’s Hospital Kilkenny: G. Courtney. Children’s Health Ireland: P. Gavin, B Freyne, C.
