# Supplementary material for: Associations between type III interferons, obesity and clinical severity of COVID-19
Source: Front Immunol. 2025 Apr 22;16:1516756. doi: 10.3389/fimmu.2025.1516756 (PMC12052549; doi:10.3389/fimmu.2025.1516756)
Supplement: Supplementary file 1 [file DataSheet1.docx]

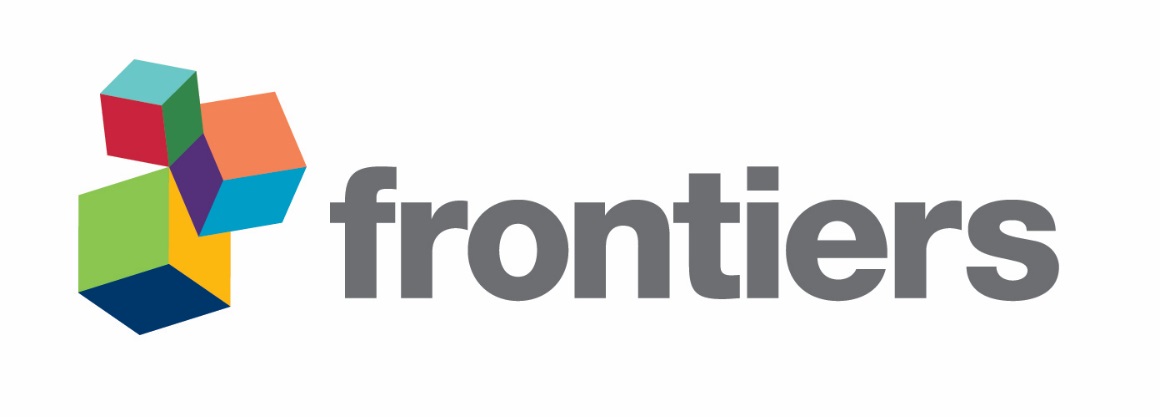


Supplementary materials for manuscript entitled “Associations between Type III Interferons, Obesity and Clinical Severity of COVID-19”.

This file includes;

- Supplementary table 1 describing Interferon λ SNPs genotype characteristics and association with COVID-19 disease severity.
- Supplementary table 2 detailing the SNP genotyping primers and probes used for the genotyping assay.
- Supplementary figure 1 detailing the frequency of IFNλ4 SNPs in comparison to the 1000 Genome project.
- Supplementary figure 2 detailing the kinetics of interferons expression following symptom onset.

Supplementary Materials

Supplementary Table 1. Interferon Lambda rs368234815 and rs117648444 SNP Genotypes Stratified for COVID-19 Disease Severity

| **IFNλ4 SNPs/**  **Outcome** | **rs368234815** | **rs117648444** | **Total**  **[n=853]** | **Mild**  **[n=500 (58.6%)]** | **Moderate**  **[n=164 (19.2%)]** | **Severe**  **[n=189 (22.2%)]** | **P** |
| --- | --- | --- | --- | --- | --- | --- | --- |
| **IFN-λ4 Null** | **TT/TT** | **G/G** | 471 (55.3%) | 271 (57.5%) | 92 (19.5%) | 108 (23.0%) | 0.9590 |
| **IFNλ4 Weak** | **TT/∆G** | **G/A** | 66 (7.73%) | 41 (62.1%) | 12 (18.2%) | 13 (19.7%) |  |
|  | **∆G/∆G** | **A/A** | 4 (0.46%) | 2 (50.0%) | 1 (25.0%) | 1 (25.0%) |  |
| **IFNλ4 Strong** | **∆G/∆G** | **G/A** | 30 (3.51%) | 20 (66.7%) | 4 (13.3%) | 6 (20.0%) |  |
|  | **∆G/∆G** | **G/G** | 38 (4.45%) | 23 (60.5%) | 9 (23.7%) | 6 (15.8%) |  |
|  | **TT/∆G** | **G/G** | 244 (28.6%) | 143 (58.6%) | 46 (18.9%) | 55 (22.5%) |  |
| **rs368234815** | **TT/TT** | | 471 (55.3%) | 271 (57.5%) | 92 (19.5%) | 108 (23.0%) | 0.7552 |
|  | **TT/ΔG** | | 310 (36.3%) | 184 (59.3%) | 58 (18.7%) | 68 (22.0%) |  |
|  | **ΔG/ ΔG** | | 72 (8.4%) | 45 (62.5%) | 14 (19.5%) | 13 (18%) |  |
|  | **TT/TT**  **Not Expressing** | | 471 (55.3%) | 271 (57.5%) | 92 (19.5%) | 108 (23.0%) | 0.6899 |
|  | **TT/∆G, ∆G/∆G**  **Expressing** | | 382 (44.8%) | 229 (60.0%) | 72 (18.8%) | 81 (21.2%) |  |
| **rs117648444** | **G/G** | | 753 (88.3%) | 437 (58.0%) | 147 (19.5%) | 169 (22.5%) | 0.8769 |
|  | **G/A** | | 96 (11.3%) | 61 (63.5%) | 16 (16.7%) | 19 (19.8%) |  |
|  | **A/A** | | 4 (0.4%) | 2 (50.0%) | 1 (25.0%) | 1 (25.0%) |  |

Legend: Interferon Lambda rs368234815 and rs117648444 SNP Genotypes Stratified for COVID-19 Disease Severity. Chi-Square χ^2^ tests to compare the frequencies between the groups.

| **NCBI**  **dbSNP ID** | **Probe Name** | **Size** | **3' Label** | **5' Label** | **Probe Sequence (5' to 3' and 11-45 bases)** | **No. of Bases** | **Probe Name** | **Cat/Part Number** |
| --- | --- | --- | --- | --- | --- | --- | --- | --- |
| [368234815](https://www.ncbi.nlm.nih.gov/snp/?term=368234815) | rs368234815_VIC_TT | 6,000 picomoles | MGB (Liquid Only) | VIC® (MGB/TAMRA/QSY®) | ATCGCAGAAGGCC | 13 | rs368234815_VIC_TT | 4316034 |
|  | rs368234815_FAM_dG | 6,000 picomoles | MGB (Liquid Only) | 6-FAM™ (MGB/TAMRA/QSY®) | ATCGCAGCGGCCC | 13 | rs368234815_FAM_dG | 4316034 |
| [117648444](https://www.ncbi.nlm.nih.gov/snp/?term=117648444) | rs117648444_C | 6,000 picomoles | MGB (Liquid Only) | VIC® (MGB/TAMRA/QSY®) | CGGAGGATCCCTCC | 14 | rs117648444_C | 4316034 |
|  | rs117648444_T | 6,000 picomoles | MGB (Liquid Only) | 6-FAM™ (MGB/TAMRA/QSY®) | CGGAGAATCCCTCC | 14 | rs117648444_T | 4316034 |
| **NCBI**  **dbSNP ID** | **Primer Name** | **Size** | **Formulation** | **Primer Sequence (5' to 3' and 11-45 bases)** | | **No. of Bases** | **Cat/Part Number** | |
| [368234815](https://www.ncbi.nlm.nih.gov/snp/?term=368234815) | rs368234815_F | 80,000 picomoles | Dry | GCCTGCTGCAGAAGCAGAGAT | | 21 | 4304971 | |
|  | rs368234815_R | 80,000 picomoles | Dry | GCTCCAGCGAGCGGTAGTG | | 19 | 4304971 | |
| [117648444](https://www.ncbi.nlm.nih.gov/snp/?term=117648444) | rs117648444_F | 80,000 picomoles | Dry | GGGCCTCACCGATGGC | | 16 | 4304971 | |
|  | rs117648444_R | 80,000 picomoles | Dry | CAGCGCAACTGCTCCTT | | 17 | 4304971 | |

Supplementary Table 2. Genotyping Primers and Probes

Supplementary Graph 1. Frequency of IFNλ4 SNPs in
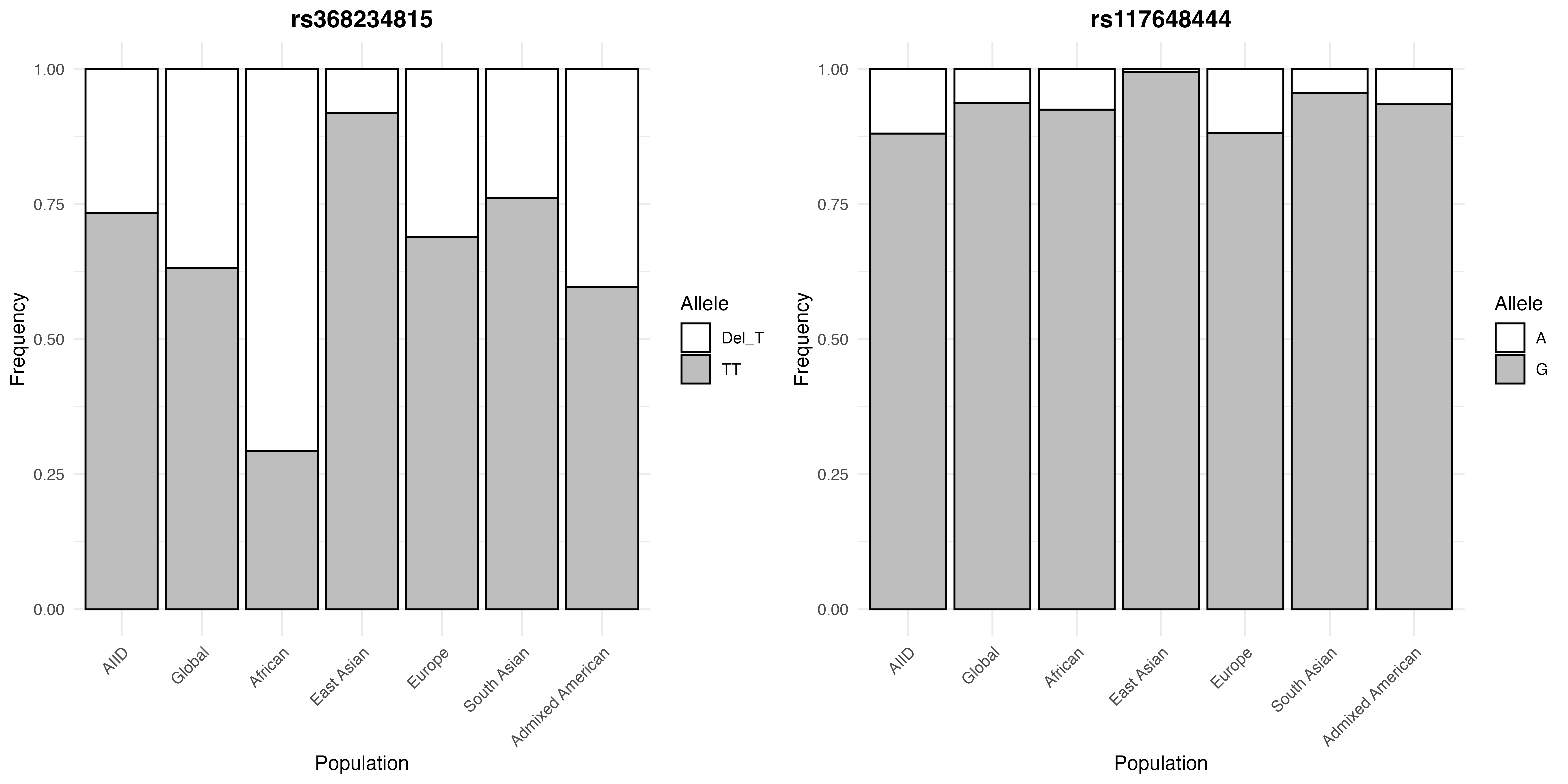
the AIID Cohort Compared to Other Populations in the 1000 Genome Project

AIID; All Ireland Infectious Disease Cohort. rs368235815 Del T includes genotypes expressing IFNλ4 (∆G/∆G and TT/∆G), while TT includes the non-expressing genotype (TT/TT). rs117648444 A includes genotypes of AA and GA, while the G only includes the GG genotype.

**Supplementary Graph 2. Kinetics of Interferon Expression Following Symptom Onset**

**
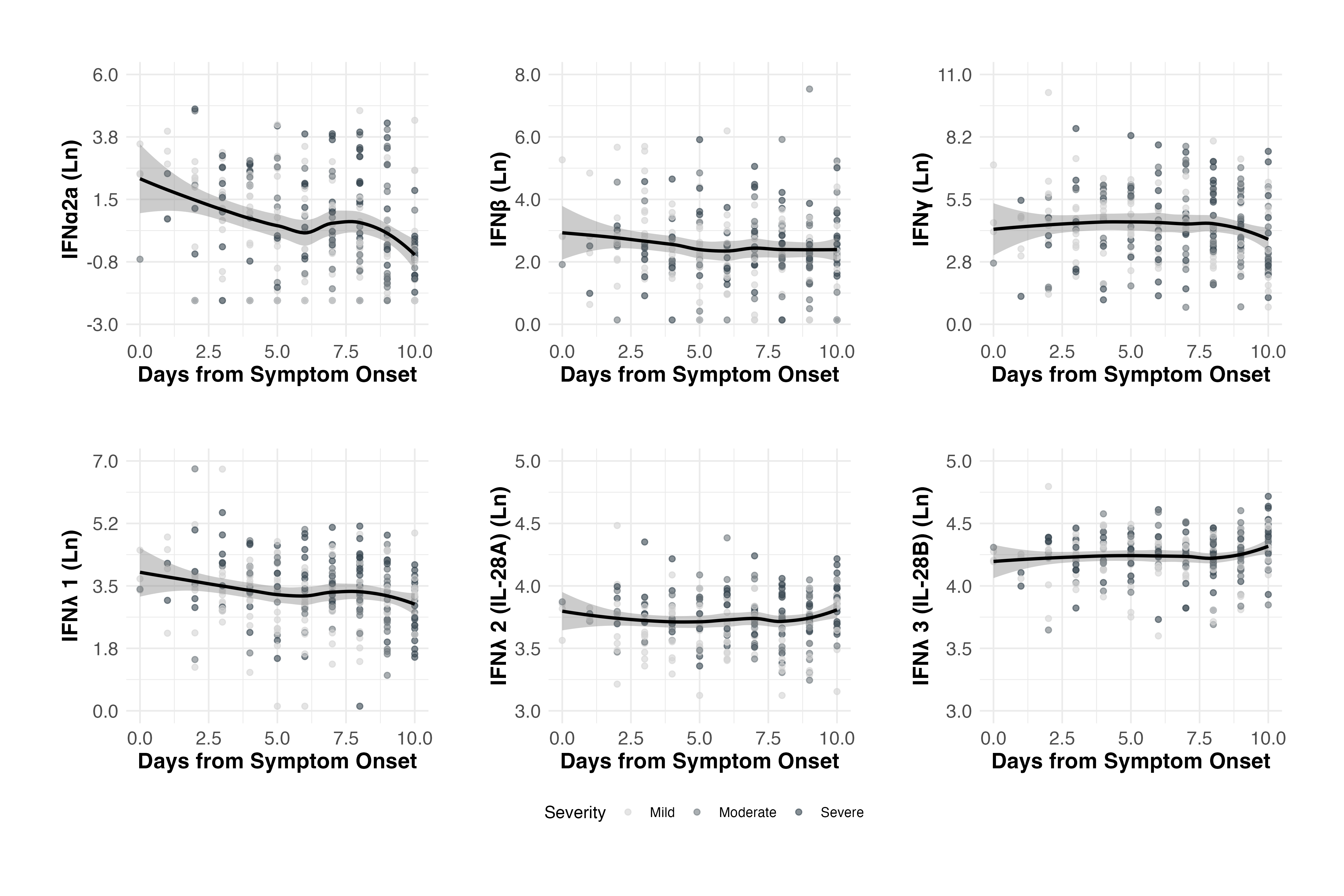
**

Ln; Natural log
